# Relation between primary tumor FDG avidity and site of first distant metastasis in patients with breast cancer: Erratum

**DOI:** 10.1097/01.md.0000503453.60850.3a

**Published:** 2016-10-07

In the article “Relation between primary tumor FDG avidity and site of first distant metastasis in patients with breast cancer”,^[1]^ which appeared in Volume 95, Issue 32 of Medicine, Dr. Chae Hong Lim's name was originally given incorrectly. The article has since been corrected online.
